# Knowledge, attitude, and practices with respect to disease surveillance among urban private practitioners in Pune, India

**DOI:** 10.3402/gha.v8.28413

**Published:** 2015-10-01

**Authors:** Revati K. Phalkey, Mareike Kroll, Sayani Dutta, Sharvari Shukla, Carsten Butsch, Erach Bharucha, Frauke Kraas

**Affiliations:** 1 Institute of Geography, University of Cologne, Cologne, Germany; 2 Division of Epidemiology and Public Health, University of Nottingham, City Hospital, Nottingham, United Kingdom; 3 Institute of Environment Education and Research, Bharati Vidyapeeth Deemed University, Pune, India; 4 Center for Modelling and Simulation, Savitribai Phule University of Pune, Pune, India

**Keywords:** knowledge-attitude-practice, private practitioners, disease surveillance, barriers and facilitators

## Abstract

**Background:**

Participation of private practitioners in routine disease surveillance in India is minimal despite the fact that they account for over 70% of the primary healthcare provision. We aimed to investigate the knowledge, attitudes, and practices of private practitioners in the city of Pune toward disease surveillance. Our goal was to identify what barriers and facilitators determine their participation in current and future surveillance efforts.

**Design:**

A questionnaire-based survey was conducted among 258 practitioners (response rate 86%). Data were processed using SPSS™ Inc., Chicago, IL, USA, version 17.0.1.

**Results:**

Knowledge regarding surveillance, although limited, was better among allopathy practitioners. Surveillance practices did not differ significantly between allopathy and alternate medicine practitioners. Multivariable logistic regression suggested practicing allopathy [odds ratio (OR) 3.125, 95% confidence interval (CI) 1.234–7.915, *p*=0.016] and availability of a computer (OR 3.670, 95% CI 1.237–10.889, *p*=0.019) as significant determinants and the presence of a laboratory (OR 3.792, 95% CI 0.998–14.557, *p=*0.052) as a marginal determinant of the practitioner's willingness to participate in routine disease surveillance systems. Lack of time (137, 55%) was identified as the main barrier at the individual level alongside inadequately trained subordinate staff (14, 6%). Main extrinsic barriers included lack of cooperation between government and the private sector (27, 11%) and legal issues involved in reporting data (15, 6%). There was a general agreement among respondents (239, 94%) that current surveillance efforts need strengthening. Over a third suggested that availability of detailed information and training about surveillance processes (70, 33%) would facilitate reporting.

**Conclusions:**

The high response rate and the practitioners’ willingness to participate in a proposed pilot non-communicable disease surveillance system indicate that there is a general interest from the private sector in cooperating. Keeping reporting systems simple, preferably in electronic formats that minimize infrastructure and time requirements on behalf of the private practitioners, will go a long way in consolidating disease surveillance efforts in the state. Organizing training sessions, providing timely feedback, and awarding continuing medical education points for routine data reporting seem feasible options and should be piloted.

Surveillance is an essential public health function that is crucial to detecting the true health status of a population (1, 2). Inaccurate estimates of disease counts may inhibit effective decision-making by policy makers with respect to the prevention or control of diseases (3, 4). Disease surveillance in India, as in many other LMICs, is rather rudimentary and several gaps exist in the current surveillance efforts. Chief amongst them is that surveillance is predominantly implemented as ‘a component’ within vertical single-disease control programs. These heavily autonomous programs are minimally flexible, preventing their adequate integration at all levels within the system in which they operate (5). Disease data collected is tailored to the need of the individual program, thus rendering it less meaningful for generalized surveillance.

India, through sheer numbers, bears a large part of the global disease burden for communicable as well as non-communicable diseases (NCDs) (5, 6). Together with maternal, perinatal, and nutritional disorders, communicable diseases constituted 38% of the total deaths in the country in 2011 (7). NCDs account for 53% of all deaths in the country (54% in the state of Maharashtra) and it is estimated that they will account for 67% of all deaths by 2020 (8). Despite the rising prevalence of NCDs, a majority of the current surveillance efforts in India focus almost exclusively on communicable diseases.

Furthermore, there are several rapidly urbanizing states in the country that face the general challenges of high social gradients, inequity, and inequality in the access to healthcare. NCD surveillance is especially indispensable in urban areas where the rise in lifestyle-related chronic diseases occurs at a much faster pace. In the absence of adequate electronic medical records and unique identifiers, the high rates of out- and in-migration make surveillance a mammoth task in these settings. In Maharashtra (where the study was conducted) over 42% of the 112 million people live in urban areas. Even so, there is no urban disease surveillance program currently in place (9).

Another major gap is the limited inclusion of the private sector in surveillance activities. Currently, surveillance data is mainly collected from public healthcare facilities in the country. Public facilities remain understaffed and chronically underfunded, face capacity overload on a daily basis, and their coverage in urban areas is poor (10, 11). In the backdrop of these infrastructural inadequacies and individual socioeconomic vulnerabilities, a large part of the urban population rely on the unregulated private sector even for primary care (12, 13). Private physicians are the preferred first contact for healthcare and provide over 80% of out-patient and 60% of all in-patient care in Maharashtra (7). Nonetheless, their involvement in current disease surveillance efforts in the state, as in the rest of the country, is restricted to infectious disease outbreak response only.

Albeit with limited success, the Revised National Tuberculosis Control Program (RNTCP) is amongst the few national disease control programs actively seeking public–private partnerships in case detection and treatment (12). The Integrated Disease Surveillance Program (IDSP) implemented in 2005 is the only national program dedicated to disease surveillance that also seeks private sector cooperation. Sentinel private reporting units are a part of the IDSP, but data reporting to date is voluntary from the private sector and mandatory from the public sector facilities. In Maharashtra, the original IDSP project implementation plan (released in 2005) aimed to include at least 15–45 private practitioners per 100,000 population (14). It was revised and the goal reduced to include at least one private practitioner per block (sub-district-level administrative unit) in 2010 due to non-compliance from the private sector (15). The situation remains grim and only 50 private laboratories and 88 private hospitals have reported data to the IDSP, compared to 1,762 laboratories and 2,303 healthcare facilities from the public sector in 2012. Only 18% of these private reporting units reported data consistently (at least 40 of the 52 weeks) in 2011, 70% in 2012, down to 47% in 2013 (16). These numbers are indicative of the low levels of participation from the private sector in routine surveillance activities even for infectious diseases.

Furthermore, alternate medicine practitioners (ayurveda, homeopathy, and unani medicine) form a major part of private healthcare providers. According to the state health systems research unit in Maharashtra, there are 62 ayurvedic and 47 homeopathic training institutions in the state that cater to regular in-patient and out-patient care. Over 63,000 ayurveda, 38,407 homeopathy and 4,079 unani medicine registered practitioners provide care in the state (17). Although the IDSP does not differentiate between allopathy and alternate medicine practitioners, these are inadequately included in other surveillance efforts.

In summary, three factors together create major gaps in the surveillance efforts in the state: a strong focus on communicable diseases, inadequate inclusion of the private sector, and finally neglect of alternate medicine practitioners. The data collected by current surveillance activities is non-representative and therefore unsuitable for decision-making. Although the forthcoming National Urban Health Mission is expected to plug some of these gaps, the hesitation of the private sector to actively participate in disease surveillance remains the greatest challenge that needs to be overcome, particularly in the fast expanding urban areas (18). As one step toward solving this problem we investigated the knowledge, attitudes, and practices (KAP) of relevance to disease surveillance among urban private healthcare providers (including alternate medicine practitioners) in Pune, Maharashtra. We identified the barriers and facilitators to their participation in current surveillance efforts and assessed their willingness to participate in future programs.

## Methods

### Study area

A questionnaire-based KAP survey was conducted in July and August 2013 in three purposively sampled areas in the city to represent the different phases of urban development. These included an inner city ward (city administrative area) with the maximum density of private healthcare facilities (Kasba-Vishrambaugwada), one ward at the urban fringe of the city (Dhankawadi), and a third rapidly developing area in the suburban fringe (Pirangut/Lavale).

### Survey sample

Private practitioners in the city consisted of allopathy (modern medicine) and alternate medicine practitioners including ayurveda, homeopathy, and unani medicine (for detailed description of the systems, please refer to www.indianmedicine.nic.in/). Since not all private practitioners are registered with their respective regulatory bodies or with the city administration, a comprehensive list of all practicing private healthcare providers within the three areas was not available. Two research fellows walked systematically through the three areas, guided by Google Maps, and plotted private facilities that provide general medical care. Facilities were mapped using a MobileMapper 6W/GIS (Ashtech by Magellan Professional, Santa Clara (California), USA) and allotted a unique identification number. Maps were generated and validated when all identified facilities were physically visited a second time during the KAP survey. All facilities (*n*=370, 100%) offering general medicine and primary care in the three areas (that operated for more than 10 h per week) were approached for a semi-structured questionnaire-based interview.

### Data collection tool

A semi-structured questionnaire (Supplementary file 1) was subjected to a two-step peer review by all researchers on the project, the state surveillance unit, and a statistician to improve content validity. After subsequent modifications, it consisted of five sections covering data on demographic information of the respondent, infrastructure at the facility (including availability of medical records and IT equipment), diagnostic routines for six selected diseases (diabetes, cardiovascular diseases, chronic respiratory diseases, cancers, tuberculosis, and dengue) and the availability of in-house laboratory facilities. Section 4 assessed the respondents’ knowledge about disease surveillance and their attitude toward data recording and reporting. Section 5 probed respondents’ interest in participating in a pilot study on NCD surveillance and investigated how data collection should be organized.

### Survey protocol

Ethical approval was obtained from the Ethics Commission of the Faculty of Medicine of Cologne University [Ethikkommission der Medizinischen Fakultät der Universität zu Köln (No. 13-107)]. The questionnaire was pre-tested in a different ward (Kondhwa) and training was conducted for all investigators. Each facility was physically visited during opening hours (as indicated on the boards outside or as noted by the respondent). If a facility declined to participate or was closed during three visits, it was excluded. Busy practitioners were asked for an appointment at the first visit. If the questionnaire could not be administered, at the second visit a modified version was left for them to fill out later. It was then collected by appointment. In facilities with more than one practitioner, only the heads of the facility (hospital administrators in large hospitals) were included in the survey. Mandatory informed oral consent was obtained from all willing participants. Parts of the questionnaire were administered in Marathi (local language) but the responses and notes were always recorded in English.

### Data entry, cleaning, and processing

Data were entered by individual investigators in a preset data entry mask using EpiData 3.1 and subjected to random quality checks. Descriptive analyses were done using Microsoft^®^ Excel 2011 and Statistical Package for the Social Sciences version 17.0.1 (SPSS™, Chicago, IL, USA). Multivariable logistic regression was carried out to look at the determinants of practitioner participation (*n*=258) in routine disease surveillance systems, which was coded as 0 for ‘willing to participate’ and 1 for ‘not willing to participate’.

The independent variables (number of years of practice, qualifications, system of medicine, and variables related to infrastructure such as electricity, computers, etc.) were coded into dummy variables. In another model (*n*=129) we included in-house diagnostics (x-ray, laboratory, spirometry, and rapid diagnostic tests).

Furthermore, a five-stage framework approach was used for qualitative data analysis from open questions (19). Raw textual data entered in SPSSTM Inc., Chicago, IL, USA, version 17.0.1 were used to list the key areas and identify recurrent themes (*familiarization*). These were then aligned to barriers and facilitators (*identifying the thematic framework*). All the textual data was *indexed* to relevant themes and rearranged to fit barriers or facilitators (*charting*). *Interpretations* were used to understand existing gaps. A general inductive approach was taken to summarize the findings from open-ended questions and informal discussions following the interviews (20).

## Results

A total of 71 of the 370 practitioners were excluded from the study because either the facility had shut down (30, 42%), it was closed during three visits (27, 38%), the practitioner was out of station (8, 11%), or because the facility was not open for more than 10 h per week (6, 8%). A total of 299 practitioners were approached for an interview, of which 258 agreed to participate (response rate 86%). The main reasons given for refusal to participate included lack of interest (15, 37%) and lack of time (5, 12%). The rate of refusal was higher in the inner city ward (29, 21%) and among postgraduate (14, 17%) and allopathic practitioners (20, 25%).

### Profile of the respondents

Table 1 shows the profile of the 258 participants, which included 59 (23%) allopathic, 111 (43%) ayurvedic, 85 (33%) homeopathic, and 3 (1%) unani medical practitioners. For the purpose of analysis we combined the data for the three unani practitioners with the ayurvedic practitioners. A majority of the respondents were males (184, 71%) and held a graduate degree (192, 74%) in their respective system of medicine. The share of postgraduate practitioners was highest (30, 51%) among allopathic practitioners.

**Table 1:** General characteristics of the respondents (*n*, %)

		Allopathy (59)	Ayurveda and Unani (114)	Homeopathy (85)	Total (*N*=258)
Location	Inner city ward	32 (54)	48 (42)	27 (32)	107 (41)
	Urban fringe ward	21 (36)	57 (50)	50 (59)	128 (50)
	Suburban fringe area	6 (10)	9 (8)	8 (9)	23 (9)
Gender	Male	45 (76)	82 (72)	57 (67)	184 (71)
	Female	14 (24)	32 (28)	28 (33)	74 (29)
Qualification	Graduate	29 (49)	96 (83)	67 (79)	192 (74)
	Postgraduate	30 (51)	18 (16)	18 (21)	66 (26)
Duration of practice	Total mean (SD)	23.1 (12.4)	14.3 (11.2)	12.0 (8.3)	15.4 (11.4)
	Inner city ward	28.1 (10.4)	18.4 (13.2)	15.3 (9.2)	20.4 (12.5)
	Urban fringe ward	17.1 (12.4)	10.6 (7.8)	10.6 (7.5)	11.7 (8.9)
	Suburban fringe area	19.2 (12.4)	12.2 (11.0)	9.1 (7.1)	12.9 (10.6)
Patients/day	Mean (SD)	25 (25)	22 (17)	12 (8)	15 (11)
Catchment area	Majority same ward	32 (54)	78 (68)	58 (68)	168 (65)

The mean duration of practice was 15.4 years (SD 11.4) and was highest in the inner city ward (20.4 years, SD 12.5), especially among allopathic practitioners. A total of 111 (44%) practitioners had less than 10 years of experience, 29% between 10 and 20 years, and 27% over 20 years. The mean number of patients visiting the facilities per day was 15 (SD 11) and was higher for the allopathic and ayurvedic practitioners compared to homeopathic practitioners. Of the respondents, 168 (65%) reported that the majority of their patients came from the same administrative ward.

### Infrastructure to support surveillance activities

A majority of the practitioners (184, 71%) practiced in small single-headed clinics without a receptionist (151, 58%). Only a few (53, 20%) practitioners offered inpatient care, the majority of these being allopathic practitioners (19, 32%). All respondents had electrical connections in their facilities; however only half (134, 54%) had access to back-up generators (Table 2). Fewer than half (101, 39%) of the facilities had computers and 115 (45%) practitioners had access to the Internet, mainly on their smartphones. There were no significant differences among the practitioners on the basis of the system of medicine they practiced.

**Table 2:** Surveillance capacities among respondents (*n*, %)

	Allopathy (59)	Ayurveda and Unani (114)	Homeopathy (85)	Total (*N*=258)
Logistics and equipment				
Electricity backup	43 (75)	51 (45)	40 (49)	134 (54)
Computer	27 (46)	44 (38)	30 (36)	101 (39)
Internet	28 (48)	48 (42)	39 (46)	115 (45)
Maintain minimum data (age, gender, diagnosis, test results)	16 (27)	19 (17)	24 (28)	59 (23)
Human resources				
Receptionist	42 (71)	38 (33)	27 (32)	107 (41)
Paramedic	21 (36)	24 (21)	10 (12)	55 (21)
Diagnostic and treatment infrastructure				
X-ray	9 (15)	5 (4)	6 (16)	20 (15)
ECG	21 (36)	21 (18)	17 (45)	59 (46)
USG	4 (7)	3 (3)	2 (5)	9 (7)
Laboratory	13 (22)	13 (11)	11 (13)	37 (14)
Spirometry	8 (14)	6 (5)	1 (1)	15 (6)
Rapid diagnostic tests	33 (56)	36 (3)	25 (29)	94 (36)
In-patient admission	19 (32)	18 (16)	16 (19)	53 (20)

### Record-keeping practices

Over 244 (95%) practitioners reported maintaining patient registers regularly. Only 29 (11%) did so in an electronic format. While a majority of them recorded the name (*n*=239, 98%) and weight of the patients (*n*=202, 83%), over 60% reported that they recorded diagnosis, gender, address, and prescription. Fewer than half recorded age (*n*=111, 45%) and referral (*n*=88, 36%), and only a third (70, 29%) assigned their own reference or unique case identification number to the patients. If we consider age, gender, diagnosis, and test results as minimal record keeping practice for disease surveillance, then only 59 practitioners (23%) maintained data for all four essential parameters.

### Diagnostic investigations

More than 90% of the practitioners reported that they diagnosed diabetes, cardiovascular diseases and chronic respiratory diseases in their clinics (Table 3), mainly through lab confirmation. More than half of the practitioners said they also treated these cases in their clinics. For dengue and tuberculosis, 75 and 73% respectively said they diagnosed cases in locus both clinically and with lab confirmations. About half (52%) of the practitioners said they did diagnose cancers in the clinics but in most cases (79%) the patients were directly referred to specialists or to tertiary hospitals on mere clinical suspicion. Although non-allopathic practitioners diagnosed and treated both communicable and NCDs, few had the necessary diagnostic infrastructure to do so (Table 2). While rapid diagnostic tests such as a glucometer for diabetes were more commonly available (36%), only 14% of all facilities had their own laboratory.

**Table 3:** Diagnostic and treatment practices (*n*, %)

		Allopathy (59)	Ayurveda and Unani (114)	Homeopathy (85)	Total (*N* = 258)
Diabetes	Diagnosis	58 (98)	109 (96)	78 (92)	245 (95)
	Laboratory confirmation	52 (90)	99 (91)	73 (94)	224 (91)
	Treatment in clinic	45 (78)	58 (53)	46 (59)	149 (61)
Cardiovascular diseases	Diagnosis	55 (93)	107 (94)	75 (88)	237 (92)
	Laboratory confirmation	44 (80)	73 (68)	55 (73)	172 (73)
	Treatment in clinic	40 (73)	50 (47)	40 (53)	130 (55)
Chronic respiratory diseases	Diagnosis	53 (90)	105 (92)	76 (89)	234 (91)
	Laboratory confirmation	40 (75)	72 (69)	53 (70)	165 (71)
	Treatment in clinic	43 (81)	63 (60)	50 (66)	156 (67)
Cancers	Diagnosis	39 (66)	52 (46)	44 (52)	135 (52)
	Laboratory confirmation	26 (67)	21 (40)	22 (50)	69 (51)
	Treatment in clinic	7 (18)	10 (19)	11 (25)	28 (21)
Tuberculosis	Diagnosis	53 (90)	76 (76)	60 (71)	189 (73)
	Laboratory confirmation	45 (85)	50 (66)	53 (88)	148 (78)
	Treatment in clinic	38 (72)	21 (28)	18 (30)	77 (41)
Dengue	Diagnosis	51 (86)	84 (74)	59 (69)	194 (75)
	Laboratory confirmation	47 (92)	59 (70)	52 (88)	158 (81)
	Treatment in clinic	35 (69)	38 (45)	27 (46)	100 (52)

### Knowledge and general awareness regarding disease surveillance

Knowledge regarding surveillance was low overall. Fewer than half of the respondents (121, 47%) were able to name at least one function of disease surveillance (Table 4) and only three (1%) were able to mention all four functions [as identified by the World Health Organization: data collection, analysis, dissemination, and application (2)]. Allopathic practitioners were generally more aware about these functions. Around one-third (36%) of the respondents could name at least two state disease control programs requiring reporting from the private sector. These included mainly the RNTCP (136, 53%) and the National Vector Borne Disease and Control Program (108, 42%). Despite their limited knowledge, a majority of the practitioners (240, 93%) agreed on the importance of disease surveillance for improving urban health. A majority (60%) of the respondents stated that government estimation of the disease burdens for both communicable and NCDs in Pune were gross underestimations of the actual numbers. This situation, almost all agreed, was because of incomplete reporting and the exclusion of the private sector.

**Table 4:** Knowledge and opinion regarding surveillance efforts (*n*, %)

	Allopathy (59)	Ayurveda and Unani (114)	Homeopathy (85)	Total (*N*=258)
Knowledge regarding disease surveillance				
Aware about disease surveillance	39 (66)	45 (39)	37 (44)	121 (47)
Surveillance components named by the practitioners				
Systematic collection of disease information	25 (42)	26 (23)	20 (23)	71 (27)
Analysis of disease information	11 (19)	13 (12)	16 (19)	40 (15)
Dissemination to allow action	9 (15)	13 (12)	8 (9)	30 (12)
Application of data for disease control	24 (41)	15 (13)	12 (14)	51 (20)
Able to name at least two national disease control programs	25 (42)	38 (33)	31 (36)	94 (36)
Opinion regarding surveillance efforts				
Current infectious disease burden is *not* adequately captured	42 (71)	61 (54)	48 (57)	151 (59)
Current NCD burden is *not* adequately captured	44 (75)	66 (58)	50 (59)	160 (62)
Disease surveillance is important for urban health	54 (93)	106 (93)	80 (95)	240 (94)

NCD, non-communicable disease.

### Practices with respect to surveillance

A total of 101 (52%) of the 194 and 103 (54%) of the 189 practitioners who diagnosed dengue and tuberculosis, respectively, said that they also reported it to the Pune Municipal Corporation (city administration). Both are mandatory reportable diseases. Of the respondents, 96% (247) agreed that the involvement of private practitioners was important to improve urban health. Only 71 (27%) of the respondents reported having been approached by a state disease control program to participate in regular surveillance activities during the previous year. Of these, 67 (94%) reportedly agreed to participate. This fact could indicate a clear lack of initiative from the national disease control agencies to include the private practitioners in their surveillance efforts.

### Attitude and willingness to participate in surveillance

Overall, 195 (76%) practitioners said that they were willing to participate in a routine sentinel surveillance system on a continuous basis. When asked specifically if they wanted to participate in a pilot sentinel surveillance system for NCDs, 180 (70%) responded positively, and 25 were undecided (10%). The majority of the practitioners suggested that such a system should focus on cardiovascular diseases (168, 65%) and diabetes (172, 67%) but should also include chronic obstructive pulmonary disease (COPD) (90, 35%) and cancer (87, 34%). Two-thirds of the practitioners (170, 85%) recommended a paper-based system with a monthly data collection cycle (157, 78%).

### Logistic regression

Multivariable logistic regression revealed that the system of medicine practiced influenced the practitioner's willingness to participate in routine disease surveillance activities, with respondents practicing allopathy more likely to respond positively [odds ratio (OR) 3.125, 95% confidence interval (CI) 1.234–7.915, *p*=0.016] compared to those practicing ayurveda or homeopathy. In the same way the availability of a computer at the facility (OR 3.670, 95% CI 1.237–10.889, *p*=0.019) was a predictor of the respondents’ willingness to participate in surveillance activities. The model comparing in-house investigations revealed that the presence of a laboratory within a facility (OR 3.792, 95% CI 0.998–14.557, *p*=0.052) was also a marginal determinant. All other parameters were non-significant, including the years of practice; minimum amount of patient information collected in registers; availability of a phone, electricity, generator backup, or overnight admission facility; record registration format; availability of a receptionist and paramedic staff; and awareness of surveillance and diagnostic capacities such as x-ray, spirometry, or rapid diagnostic tests.

### Barriers and facilitators

In total, 219 (85%) of all respondents identified at least one facilitator or barrier for participation in regular surveillance activities (Table 5). Lack of time (55%), lack of motivation or routine (9%), and lack of infrastructure (6%), that is, trained staff and equipment, for completing necessary paperwork for reporting surveillance data were the most frequently reported barriers at the individual level. Problems with patient interaction, that is, confidentiality of patient data, lack of follow-up visits, and missing lab confirmations due to high costs, were mentioned by 11% of the respondents as further barriers to participation in surveillance.

**Table 5:** Barriers and facilitators for participation in regular surveillance (multiple answers possible) (*n*, %)

Barriers (*N*=251)		Facilitators (*N*=195)	
Intrinsic (individual/facility level)			
Lack of time	137 (55)	Monetary/infrastructural incentives	33 (17)
Attitude: lack of motivation/routine	23 (9)	Acknowledgment of efforts	10 (5)
Lack of infrastructure (personal/equipment, etc.)	14 (6)	Participation in research	6 (3)
Patient interaction: follow-up with patient, data privacy	11 (4)		
Extrinsic (government level)			
Poor cooperation government/private sector	27 (11)	Information/awareness/training about surveillance	78 (40)
Legal issues with alternate medicine	15 (6)	Mandatory regulations	30 (15)
Effective use of collected data	11 (4)	Better reporting system	25 (13)
Lack of clear guidelines for submitting data	7 (3)	Others	13 (7)
Others	6 (2)		

Major extrinsic barriers included lack of cooperation between the government and private sector (27, 11%) and legal issues (15, 6%) with reporting data as alternate medicine practitioners. One of the ayurvedic practitioners mentioned the fear of legal consequences such as license revocation for mismanagement of cases or prescription of allopathic drugs (which such practitioners are not formally qualified to administer) if they submitted data on a tuberculosis or dengue case. One of the practitioners described problems regarding timely reporting of cases as follows: ‘If a general practitioner reports a case, the PMC [Pune Municipal Corporation] asks directly why the report is two, three days late, but lab confirmation requires time and often the patient does not go for lab confirmation due to lack of money or awareness, so the general practitioner doesn't get case confirmation. They do not accommodate these issues’.

As for the incentives, a majority (78, 40%) of the respondents felt that providing them with more information about disease surveillance, practices, and processes would prove beneficial and help to improve case reporting. Out of these, eight practitioners (4%) suggested training through continuing medical education (CME) as an option. Other respondents suggested monetary and infrastructural (e.g. software, computer) incentives (33, 17%), making reporting a mandatory activity (30, 15%), and an improved reporting system with clear and simple guidelines (25, 13%) as useful measures to improve reporting from the private sector.

## Discussion

The study is valuable in identifying a number of issues with respect to urban disease surveillance and helps to clear some prevailing misconceptions. The choice of three distinct geographical areas within the same agglomeration helped to develop an understanding of how areas in different stages of urban development dealt with the challenge of primary healthcare provision. Patterns that emerged (e.g. higher amount of allopathic, postgraduate, and more experienced practitioners in the inner city center), although not surprising, were a first of its kind quantifications in the agglomeration and hence valuable. The study indicates that the private sector plays an important role in initial screening, diagnosis, and treatment for both communicable and NCDs. Private practitioners of all systems of medicine serve as essential primary healthcare providers in the city. The involvement of private practitioners including ayurvedic and homeopathic practitioners in routine disease surveillance systems is therefore crucial and must be adequately addressed in the upcoming National Urban Health Mission implementation plans. The study was also successful in validating several known barriers to private sector participation in routine surveillance activities that need to be addressed in future programs.

### Formalizing knowledge and improving understanding

Few respondents were able to correctly describe surveillance, its importance, or its components. However, most of them (60%) were confident (based on their personal observations) that the disease burden and distributions in the city as captured by the administration are inaccurate. When asked to name the national disease control programs, respondents struggled. For several of them it was a surprise that these programs contribute to ‘surveillance’. This situation strongly indicates the need for setting up formal training on surveillance needs, objectives, processes, and outcomes.

A better understanding and knowledge with respect to surveillance among the allopathic practitioners, postgraduate degree holders, and senior practitioners indicates that the undergraduate university curricula for ayurveda and homeopathy need revision. In view of the general willingness from all stakeholders, one solution could be an annual mandatory local CME session on disease surveillance. Ideally, these sessions should be conducted by the Indian Medical Association and organized by the state/city surveillance office. They should bring together the private sector regulating body (although only voluntary) and the city administration. Initiating a dialogue between stakeholders, including similar regulatory bodies for ayurvedic and homeopathic practitioners, should be the next step.

### Improving surveillance infrastructure and hence the practices

The constraints of awareness with respect to surveillance were reflected in the respondents’ data-keeping practices. Although a relatively high number indicated that they kept patient records of some sort, very few (59, 23%) maintained records for age, gender, diagnosis, and test results. These records are considered minimal data requirements, besides the location of the case, for surveillance purposes. Similarly, only half of the respondents admitted to reporting even the mandatory notifiable diseases (tuberculosis and dengue) to the municipal corporation (city administration). Even this number may be an overestimation as some responses may be the result of a social desirability bias. Training and awareness building are most likely the only option to improve these aspects of the surveillance practices (21, 22). In addition, regular feedback, supervision, and involvement of the practitioners in the decision-making processes will encourage ownership and prove beneficial when formulating a system in the future (23).

Second, most facilities in the city are small clinics with single practitioners. Minimal infrastructure (human resources, computers, and diagnostic capacities) especially among alternate medicine practitioners was identified as the main hindering factor for willingness to participate in surveillance activities. Time-consuming reports coupled with complicated and resource-intensive reporting procedures are known to jeopardize data reporting from the private sector (24, 25). Therefore, developing a simple system with clear operating procedures is the key. Additionally, a majority of the practitioners still prefer a paper-based system with monthly data collection. However, given that a significant number of the medical practitioners had smartphones, their use for surveillance activities could be explored. Successful use of mobile reporting has been documented in Tanzania (26) and was also useful during the swine flu epidemic in Pune (although only verbal reporting). Lessons learned from these examples could be useful and should be explored. In the long term, the implementation of a standardized electronic medical record system has to be considered (27, 28). This would be time efficient and help improve data processing. However, given the basic infrastructure in these small clinics, we will have to remember that the implementation of such an approach would be seen as time and resource intensive.

### Addressing the challenges to case detection and reporting

Except for cancers, the majority of the diseases were suspected, diagnosed, and treated by practitioners from all systems of medicine. Confirmation of diseases by specialist opinion (presumptive diagnosis) or supported by laboratory investigations is primarily dependent on the financial capability of the patient. While diabetes and hypertension are easier to confirm at the primary level through the use of glucometers and sphygmomanometers, confirmation of COPD or dengue, for example, require clinical examination by specialists supported by advanced laboratory investigations. Furthermore, whether appropriate ‘case definitions’ were applied for disease diagnosis and classification remains uncertain especially among non-allopathic practitioners who have their own methods for diagnosis and disease classification. The treatment of cases is often based on clinical diagnosis alone, and the dispensing of allopathic drugs by alternate medicine practitioners to potentially inaccurately diagnosed cases is a matter of concern. This also explains the hesitation of alternate medicine practitioners to report these cases (because they are diagnosed mainly on the basis of clinical suspicion alone) to surveillance systems (especially sensitive diseases such as tuberculosis). A practical solution to this issue would be to enable these practitioners to diagnose, treat, and hence report these cases accurately by offering formal recognition of their contributions and further training. Again the stress should lie on employing an all-inclusive approach for training and CMEs for urban healthcare practitioners irrespective of the system of medicine they practice.

### Managing the expectation mismatch

The disconnect between the state/city administration and the private sector was clearly evident in the survey. Although only one-third of the participants were approached by any state disease control program for participation in surveillance and disease control activities during the last 12 months, over two-thirds of those approached agreed to assist. The high response rate in our study as well as the willingness of the practitioners to participate in the proposed pilot NCD surveillance system (although it may be an overestimation due to high social desirability bias) also indicates that there is an interest on the part of the private practitioners to participate. However, against the backdrop of legal powers that the state exercises in the licensing and registration of clinics and the difficulties faced by the alternate medicine practitioners in diagnosing, treating, and reporting diseases, there is higher hesitation among the practitioners to participate in surveillance activities (29). When we contacted representatives of the medical practitioners’ voluntary organization (the Indian Medical Association), they were skeptical of any partnerships and believed that the private sector was used as a scapegoat when outbreaks occurred. They were often accused of not reporting cases ‘in time’ given that they are the first point of contact for care for many patients and therefore in the best position to raise an alarm for an impending outbreak. This also resonated in the informal discussions with participants of the study as well as the state officials. This indicated a clear mismatch in expectations of both the government and the private sector. The city and state authorities recognize the role and share of the private sector in urban healthcare provision, including the alternate medicine practitioners as well as their problems in case reporting. However, limited attempts have been made to solve the problem of case reporting. Finger-pointing for underreporting when disease outbreaks occur is a counterproductive activity creating further barriers to any coherent surveillance exercise.

### How to proceed?

Most of the barriers identified are intrinsic to the private facilities and may require focused individual-level efforts; however at the same time external facilitators from the administration and clear legal frameworks will be required in the long term. Several barriers at the policy level hinder existing and future surveillance efforts. We could find only gross estimates of how many clinics operate in a ward. In the absence of accurate denominators, any effort in collecting surveillance data is compromised. A unique registration platform to capture and regulate all private healthcare providers irrespective of their system of medicine (including their degree and qualification) in an urban area is urgently required before any effective surveillance effort can be established. Second, a legal framework dealing with surveillance should be established. This must include diagnoses and treatment of both communicable and NCDs by alternate practitioners with allopathic drugs. It should probably also consider obligatory reporting of notifiable diseases from the private sector against the current ‘voluntary only’ approach. Mandatory recording and reporting from the private sector for selected diseases has been recommended and already successfully implemented (30). It worked well in Pune during the 2009 swine flu epidemic. Although it may take time to be formulated and acceptance of the system by the practitioners is not guaranteed, this option has to be explored.

Another feasible but challenging solution would be to allot CME points to practitioners participating in routine surveillance systems and reporting data consistently (e.g. more than 40 weeks in a year). Such a system would improve the motivation of the practitioners to systematically and regularly report cases. Pilot testing of this approach even for experimental purposes might be useful. Finally, simple reporting structures with regular feedback and occasional supervision would facilitate data completeness and quality (23, 24). Mechanisms and institutional structures should be, therefore, at the heart of any future urban surveillance design (24).

### Limitations of the study

The study was conducted in three different purposively selected areas of the city. Although this provides useful information, the findings may be less representative for the city as a whole or for other urban areas in the state. Three researchers and three research assistants conducted the interviews and interviewer bias cannot completely be ruled out. Responses to some questions may be a result of social desirability bias. It was difficult to map all clinics in the three areas; therefore it is possible that not all clinics were found. We interviewed only the head of the facility in clinics with more than one practicing doctor.

## Conclusions

In conclusion, there is unanimous agreement among the important stakeholders that urban disease surveillance needs strengthening and that private practitioners (including alternate medicine) should be well integrated in future systems. Although the current knowledge among the practitioners is inadequate, their overall positive attitude and willingness to cooperate is clearly evident. Efforts from the city/state authorities should be directed toward developing simplified reporting mechanisms (preferably electronic formats) while providing clear guidelines and reporting procedures. Organizing CMEs to strengthen practitioner knowledge and awarding CME points to those who report cases regularly appear as two pragmatic and feasible solutions and should therefore be piloted in the city.

## Supplementary Material

Knowledge, attitude, and practices with respect to disease surveillance among urban private practitioners in Pune, India
