# Supplementary material for: Knowledge, attitude, and practices with respect to disease surveillance among urban private practitioners in Pune, India
Source: Glob Health Action. 2015 Oct 1;8:10.3402/gha.v8.28413. doi: 10.3402/gha.v8.28413 (PMC4592845; doi:10.3402/gha.v8.28413)
Supplement: Knowledge, attitude, and practices with respect to disease surveillance among urban private practitioners in Pune, India [file GHA-8-28413-s001.pdf]

Urban Disease Surveillance

Role of Private Health Care Providers in Pune

University of Cologne

Consent Form

Purpose

The main objective of the project on urban disease surveillance in Pune is to develop and implement a prototype urban disease surveillance system which addresses the gaps identified in existing surveillance systems in Maharashtra notably weak private sector integration and exclusion of non-communicable diseases from routine surveillance. The aim is to design a generic “blueprint” which can then be extrapolated to other cities. The system will be designed in a way that it does not duplicate existing programmes but rather supplements them.

A **pilot test** will be conducted for 12 months, beginning from November 2013, within selected health care facilities in three areas in Pune (Kasba Peth, Dhankawadi and Lavale). The pilot test will help to gain important insights into routine disease surveillance. It will also provide information about opportunities and barriers for the integration of private health care facilities into routine data collection. The data analysis and the process evaluation of the pilot will help to generate recommendations on 1) how the design of the health monitoring system can be extrapolated to Pune city and 2) how components of the design could be integrated into existing surveillance structures.

This research is supervised by Prof. Dr. Erach Bharucha, Bharati Vidyapeeth University (020-24375684), and Prof. Dr. Frauke Kraas, University of Cologne, Germany (0049-221-4707050).

Voluntary Participation and Confidentiality

You have been selected as a private practitioner in one of three research areas to be part of this survey. You will be asked some questions about your clinic and your opinion regarding disease surveillance. The interview will last approximately 15 minutes.

Your participation is voluntary and you can withdraw from the survey at any point after having agreed to participate. You are free to refuse to answer any question. The information you provide is confidential and will only be used for research purposes. If I have your oral consent can I begin the interview?

Agreed: ☐1 yes ☐2 no; if no please state reason: ____
☐3 agreed to fill out form manually (if appointment not possible)
Date Visit 1: _ _/_ _ /2013 (facility closed/no time)
Date Visit 2: _ _/_ _ /2013 (facility closed during opening hours/no time)
Date Visit 3: _ _/_ _ /2013 (facility closed during opening hours/no time)

Facility ID: _ _ _/_ Investigator: ________

Respondent:

Name: ______________________________ Duration of practice (years):_____

Gender: ☐1 male ☐2 female

Qualification: ______________________ Designation: ______________________________

Speciality: ________________________ System of medicine:________________________

Phone-Nr.: ________________________ Email-ID: _________________________________

Facility infrastructure		
1	Since when is the **facility** functional?	Year: ______ ☐99 don't know
2	Does the facility have a functioning **phone** (landline or cell phone) at all times?	☐1 yes<br>☐2 no
3	a) Does the facility have **electricity** at all times?<br>b) Do you have a generator backup?	☐1 yes *(go to 3b)* ☐2 no *(go to 4)*<br>☐1 yes ☐2 no
4	Does the facility have a functioning **computer**?	☐1 yes ☐2 no
5	Is there access to **internet** within the facility today?	☐1 yes ☐2 no
6	Do you maintain **patient registers or record files** at the facility?	☐1 yes, always ☐2 yes, sometimes<br>☐3 no *(go to 16d)* ☐98 no answer
	a) If **yes**, what format?<br>*(check where applicable)*	☐1 electronic<br>☐2 paper<br>☐3 electronic and paper
	b) Which patient data do you collect on a routine basis?<br>*(check where applicable)*	☐1 name ☐2 address<br>☐3 age ☐4 gender<br>☐5 occupation ☐6 weight<br>☐7 height ☐8 case number<br>☐9 prescription ☐10 referral<br>☐11 diagnosis ☐12 test results<br>☐97 other (*pls. specify*): ____________<br>____________
	c) For how many years does this register contain data?	________ *(number of years)*
	d) If **no**: What are the main reasons? *(don't read out)*<br>*(check where applicable)*	☐1 lack of time<br>☐2 not significant<br>☐3 not mandatory<br>☐4 lack of infrastructure<br>☐97 other: ____________<br>☐98 no answer ☐96 not applicable
**7**	a) Does this facility have a receptionist?<br>b) Does this facility have paramedic or any other staff?	☐1 yes ☐2 no<br>☐1 yes ☐2 no<br>☐97 other staff: ____________
	c) For polyclinics/nursing homes/hospitals:<br>How many general practitioners and specialists are working in this facility?	☐1 no. of GPs: _____<br>☐2 no. of specialists: ____<br>☐99 don't know ☐96 not applicable
8	What is the average **number of patients** you see per day?	__________ ☐99 don't know
9	From which **areas** do the majority of your patients come from?<br>☐1 this ward *(specify area)*: ____________<br>☐2 other wards/areas: ____________<br>☐98 don't know	
10	According to you, what is the **socioeconomic background** of majority of the patients?	☐1 below poverty line (BPL)<br>☐2 lower middle class<br>☐3 middle class<br>☐4 upper middle class<br>☐5 upper class<br>☐99 don't know
11	Does the facility **admit patients overnight**?	☐1 yes: how many beds? ____<br>☐2 no

	**Diagnostics and referrals**	
12	Does this facility **diagnose** and/or refer the following diseases?	Diagnosis → (*if yes*) How are you diagnosing? → Do you treat at your facility or refer?
	a) Diabetes	□2 no □1 yes □3 clinical symptoms □4 lab-confirmed □6 treat □5 refer
	b) BP/Cardiovascular diseases	□2 no □1 yes □3 clinical symptoms □4 lab-confirmed □6 treat □5 refer
	c) Chronic respiratory diseases	□2 no □1 yes □3 clinical symptoms □4 lab-confirmed □6 treat □5 refer
	d) Cancers	□2 no □1 yes □3 clinical symptoms □4 lab-confirmed □6 treat □5 refer
	e) Tuberculosis	□2 no □1 yes □3 clinical symptoms □4 lab-confirmed □6 treat □5 refer
	f) Dengue	□2 no □1 yes □3 clinical symptoms □4 lab-confirmed □6 treat □5 refer
13	a) Does this facility provide in-house investigations? *(check where applicable)*	□1 X-ray □2 ECG<br>□3 USG □4 laboratory<br>□5 spirometry □6 rapid diagnostic test<br>□7 none

	**Disease Surveillance**	
14	Are you aware of disease surveillance?	□1 yes □2 no *(go to 16)* □3 undecided
15	How would you describe the **main functions of a disease surveillance** system? *(don't read out)*<br>□1 systematic **collection** of disease information<br>□2 **dissemination** to allow action (outbreak)<br>□3 **application** of collected data for disease prevention & control<br>□4 **analysis** of disease information<br>□5 other: ____<br>□98 don't know	
16	Do you agree that **diseases surveillance** is important to improve urban health? *(pls. add comments below)*<br>………………………………………………………………<br>…..…………………………………………………..…<br>………………………………………………………………	□1 yes □2 no<br>□3 undecided □4 no opinion
17	Do you agree that the current burden of **infectious diseases** in Pune is adequately captured?	□1 yes □2 no<br>□3 undecided □4 no opinion
18	Do you agree that the current burden of **non-communicable diseases** in Pune is adequately captured?	□1 yes □2 no<br>□3 undecided □4 no opinion
19	a) Are you aware of any **disease surveillance programs** in Maharashtra currently requiring reporting from private sector? (*don't read out*)	□1 yes *(which?)* □2 no<br>□3 IDSP<br>□4 NVBDCP (Vector Borne)<br>□5 RNTCP (TB)<br>□6 NPEP (Polio)<br>□97 Other: __________________
	b) Are you aware of the following disease control programs in your state? (*read those not checked*)	□1 Integrated Disease Surveillance Program<br>□2 Nat. Vector Borne Disease Control Program<br>□3 Nat. Revised Tuberculosis Control Program<br>□4 National Polio Eradication Program
20	a) Do you report dengue cases to the PMC?<br>b) Do you report TB cases to the PMC?	□1 yes □2 no □96 not applicable<br>□1 yes □2 no □96 not applicable

21	In your opinion, is the involvement of **private practitioners** in disease surveillance necessary to improve urban health?	□$_1$ yes □$_2$ no<br>□$_3$ undecided □$_4$ no opinion
22	Have you been **approached by any disease surveillance program** to participate in regular surveillance in the last one year?	□$_1$ yes<br>□$_2$ no *(go to 23)*<br>□$_{98}$ no answer
	a) If yes, which program?	________________________________
	b) Did you accept to be part?<br>……………………………………………………………<br>……………………………………………………………	□$_1$ yes<br>□$_2$ no<br>□$_{98}$ no answer
23	In general, would you be willing to **participate** in a routine sentinel surveillance system on a continuous basis?	□$_1$ yes □$_2$ no □$_3$ undecided
24	In your opinion, which **non-communicable diseases** should ideally be included in such a system? *(don't read out)*	□$_1$ cardiovascular diseases<br>□$_2$ cancers<br>□$_3$ chronic respiratory diseases<br>□$_4$ injuries<br>□$_5$ mental health<br>□$_6$ metabolic diseases/diabetes<br>□$_7$ none<br>□$_{97}$ others:________________________
As part of the project a pilot study will be conducted in three areas of Pune city. Data on selected diseases will be collected from private practitioners over a period of 12 months. The participation in the pilot will be entirely voluntary and can be terminated at any time during the process.		
25	Would you be **interested to participate** in our pilot study?	□$_1$ yes *(go to 25 b/c)*<br>□$_2$ no *(go to 25a, skip 26/27/28)*<br>□$_3$ undecided *(go to 25a)*
	a) If **no**, could you state some of the main reasons why you hesitate?<br>………………………………………………………………………………………………………<br>………………………………………………………………………………………………………	
	b) If **yes**, how would you prefer to submit data?	□$_1$ paper based □$_{99}$ don't know<br>□$_2$ electronically (Email)
	c) How frequent should such data be collected?	□$_1$ weekly □$_2$bi-weekly<br>□$_3$ monthly □$_{99}$ don't know
26	In your opinion is it feasible to collect the following **spatial and socioeconomic data** in regular sentinel surveillance system from private practitioners?<br>a) age □$_1$ yes □$_2$ no □$_{99}$ don't know<br>b) gender □$_1$ yes □$_2$ no □$_{99}$ don't know<br>c) address (sub ward level or pin code) □$_1$ yes □$_2$ no □$_{99}$ don't know<br>d) highest educational degree □$_1$ yes □$_2$ no □$_{99}$ don't know<br>e) occupation □$_1$ yes □$_2$ no □$_{99}$ don't know<br>f) income (broad categories) □$_1$ yes □$_2$ no □$_{99}$ don't know<br>g) BPL status □$_1$ yes □$_2$ no □$_{99}$ don't know<br>h) residency: slum/non-slum □$_1$ yes □$_2$ no □$_{99}$ don't know	
27	What **incentives** could be given to improve private practitioner participation in routine surveillance?<br>………………………………………………………………………………………………………<br>………………………………………………………………………………………………………<br>………………………………………………………………………………………………………	
28	What main **barriers** should be eliminated to facilitate private practitioner participation?<br>………………………………………………………………………………………………………<br>………………………………………………………………………………………………………<br>………………………………………………………………………………………………………	

Interviewer observations:

Interviewee Comments:
